# The Concentration of β-Carotene in Human Adipocytes, but Not the Whole-Body Adipocyte Stores, Is Reduced in Obesity

**DOI:** 10.1371/journal.pone.0085610

**Published:** 2014-01-08

**Authors:** Martin Östh, Anita Öst, Preben Kjolhede, Peter Strålfors

**Affiliations:** Department of Clinical and Experimental Medicine, Linköping University, Linköping, Sweden; National Institute of Nutrition, India

## Abstract

We have examined the concentration of β-carotene in the fat of isolated abdominal subcutaneous adipocytes obtained from lean (BMI<23 kg/m^2^), non-obese with higher BMI (23≤BMI<28 kg/m^2^), obese (BMI≥28 kg/m^2^), and from a group of obese subjects with type 2 diabetes. The concentration of β-carotene was 50% lower in the adipocytes from the obese and obese/diabetic groups compared with the lean and non-obese groups. Interestingly, the total amount of β-carotene in the adipocyte stores of each subject was constant among all groups. Triacylglycerol constituted 92±1% (by weight) of the adipocyte lipids in the lean group and this was increased to 99±2% in the obese group with diabetes (p<0.05). The concentration of cholesteryl esters was in all cases <0.1 g per 100 g of total lipids, demonstrating that mature human adipocytes have negligible stores of cholesteryl ester. Our findings demonstrate that adipocyte concentrations of β-carotene are reduced in obese subjects. The lower concentrations in adipocytes from subjects with type 2 diabetes apparently reflect subjectś obesity. Our finding that whole-body stores of β-carotene in adipocytes are constant raises new questions regarding what function it serves, as well as the mechanisms for maintaining constant levels in the face of varied adipose tissue mass among individuals over a period of time.

## Introduction

Carotenoids such as β-carotene are essential dietary constituents as humans cannot synthesize them *de novo* [1]. Following absorption of carotenoids they are transported in chylomicrons to adipose tissue and in chylomicron remnants to the liver, their main storage sites [1]–[3]. From liver they can be further distributed to VLDL [4] and LDL particles [5]. The carotenoids have two major functions, as precursors in the synthesis of retinoids and as antioxidants. Out of the several hundred existing carotenoid pigments, only a few (about ten) are detectable *in vivo* in human tissues. β-carotene is the main carotenoid and the main substrate for the synthesis of retinoids (retinaldehyde, retinol and retinoic acid) [6]. Retinoids are implied as regulators in both metabolic and mitogenic control in cells and tissues, and the underlying mechanisms are subject of intensive research. [7].

In humans the main site of carotenoid and β-carotene storage is the adipose tissue [8]. Within the adipocyte, carotenoids are stored with triacylglycerol in the lipid droplet [9]. As in case of fatty acids, CD36-expression appears to facilitate uptake of carotenoids by adipocytes [10]. The most important enzymes in the initiation of retinoid synthesis from β-carotene, β-carotene monooxygenase-1 (BCMO1) and β-carotene dioxygenase-2, are highly expressed in adipocytes [11]. It is not known to what extent β-carotene mobilization from the adipose tissue has a role in regulation of whole-body metabolism or if the adipocyte β-carotene just serves as a substrate for local usage by the adipocytes. In fact, it is not known if β-carotene is mobilized from the adipocytes. However, it is well known that also human adipocytes release the β-carotene metabolite retinol in complex with retinol-binding protein-4 (RBP4) into the circulation [12].

Studies of β-carotene in humans have mainly focused on variations in plasma levels and not much is known about the variations in tissue levels. Large population-based, epidemiological studies have established that concentrations of plasma β-carotene are inversely correlated with the measures of obesity [13], [14], markers of inflammation [15]–[17], measures of insulin-sensitivity [18], and the metabolic syndrome [18], [19]. Moreover, plasma levels of β-carotene are significantly lower in patients with type 2 diabetes compared with non-diabetic subjects [14], [20]. Studies of β-carotene in human adipose tissue and isolated adipocytes, in particular, are limited, however, obese subjects have been found to have reduced amounts of adipose tissue β-carotene compared with lean subjects [21], [22]. Also adipose tissue levels of β-carotene have been found to be higher in female compared with male subjects [23]. The extent to which adipose tissue levels reflect storage in the adipocytes and not in other cells, which account for two thirds of the adipose tissue cells, has not been assessed. One study has found differences in subcutaneous adipocytes from different anatomical locations of the same subject [24].

We measured how the content of β-carotene in isolated adipocytes varied with obesity and diabetes, and herein report that adipocytes from lean or non-obese subjects contained higher concentrations of β-carotene than cells from obese subjects, and there was no difference between cells from non-diabetic and equally obese type 2 diabetic subjects. Interestingly, total body content of adipocyte β-carotene was comparable among all groups. Moreover, the fraction of triacylglycerol of adipocyte lipids increased in obesity.

## Materials and Methods

### Ethics Statement

The study was approved by the Regional Ethics Board at Linköping University; all patients obtained written information and gave their informed verbal approval before the surgery. Verbal approval was considered sufficient by the ethics board considering that a small piece of adipose tissue was obtained during elective surgery for other reasons, and that the procedure posed no discomfort or threat to the health of the patients. The procedure was documented as part of the surgical protocol and adipose tissues samples were anonymized.

### Subjects

Subcutaneous adipose tissue was obtained from non-diabetic women (n = 43) and female patients with type 2 diabetes (n = 12), undergoing elective surgery (University Hospital of Linköping, Sweden). Subjects were considered non-diabetic, unless diagnosed with type 2 diabetes. Criteria for obesity were based on body mass index (BMI). Subjects were divided into four groups with respect to BMI and type 2 diabetes: one lean group (BMI<23 kg/m^2^) and one non-obese group with higher BMI (23≤BMI<28 kg/m^2^), one obese group (BMI≥28 kg/m^2^), and one group of obese subjects with type 2 diabetes.

Percent body-fat was calculated according to Deurenberg et al. [25] using BMI, age and sex. The calculation used is considered to overestimate the body-fat in subjects with very high BMI-values (BMI>41 kg/m^2^), however, this error is equal to the error in other methods for estimation of body fat, such as bioelectrical impedance or measurements of skinfold thickness [25]. Total adipocyte stores of β-carotene, expressed in mole per subject, were calculated from β-carotene concentration in adipocytes and total amount of body fat.

Clinical parameters such as plasma triacylglycerols, HDL/LDL- and total cholesterol along with fasting values of glucose and insulin were determined from venous blood samples taken in connection with the surgical excision of the adipose tissue. Insulin resistance index HOMA (homeostasis model assessment) was calculated from fasting plasma concentrations of glucose and insulin [26].

### Isolation of Human Adipocytes

Adipocytes were isolated by collagenase digestion (Type I, Worthington, NJ, USA) as described in [27] and washed in supplemented Krebs Ringer solution as described in [28], hence stromal vascular cells, including preadipocytes, were removed. The cells were incubated overnight in the same solution mixed with DMEM containing 7% albumin, 200 nM phenylisopropyladenosine, 20 mM Hepes, 50 UI/ml penicillin, 50 µg/ml streptomycin, pH 7.40, at 37°C. Before final analysis, cells were washed and preincubated in the Krebs Ringer solution supplemented with final concentration of 100 nM phenylisopropyladenosine, 0.5 U**·**mL^−1^ adenosine deaminase for 20 min.

### Analysis of β-carotene in Isolated Adipocytes

Isolated adipocytes were disrupted by sonication and following centrifugation, the floating lipid phase was collected for estimation of the concentration of intracellular β-carotene. Extracted adipocyte lipids were dissolved in hexane and the concentration of β-carotene was determined by HPLC-UV. Samples were diluted with 2-propanol, and aliquots were injected into the HPLC system. HPLC was performed with an HP 1100 liquid chromatograph (Agilent Technologies, Palo Alto, CA, USA), utilizing an HP1100 diode array detector set to 453 nm. Carotenoids were separated on a 4.6 mm×150 mm C30 column with 3 µm particles (YMC, Japan). The column temperature was 45°C. A two-point calibration curve was based on analysis of calibrators with known beta-carotene concentration. The limit of detection was 0.1 µM (RSD: 4%). The concentration of β-carotene is expressed per gram of triacylglycerol (TAG), thus reducing a potential problem of variations in adipocyte cell size.

### Analysis of Triacylglycerol and Cholesteryl Ester in Isolated Adipocytes

Extracts were dissolved in hexane and analyzed with an Agilent 1100 normal phase liquid chromatography system using an Evaporative Light Scattering Detector (ELSD). Separation was performed on a Chromolith Performance Si 100-4.6 mm HPLC column from Merck using hexane with MTBE and acetic acid as mobile phase. Analytes (TAG and cholesteryl esters) were calibrated against known standards from Larodan (Malmö, Sweden).

### Statistical Analyses

Statistical analyses, normality distribution testing (Shapiro-Wilks test), correlation analysis with linear regression and One-way analysis of variance (ANOVA), including post hoc testing (Bonferroni), were performed using GraphPad Prism (Version 5, GraphPad Software Inc., San Diego, CA, USA). Results are presented as means with their respective standard deviation (SD) or error (SEM), as indicated. Measured β-carotene concentrations and the calculated total body content of β-carotene were normally distributed within the groups of lean, non-obese, obese and diabetic subjects, as determined by statistical normality testing (Sharipo-Wilks test), allowing parametrical statistical methods for comparison between groups.

## Results and Discussion

**Figure 1:**
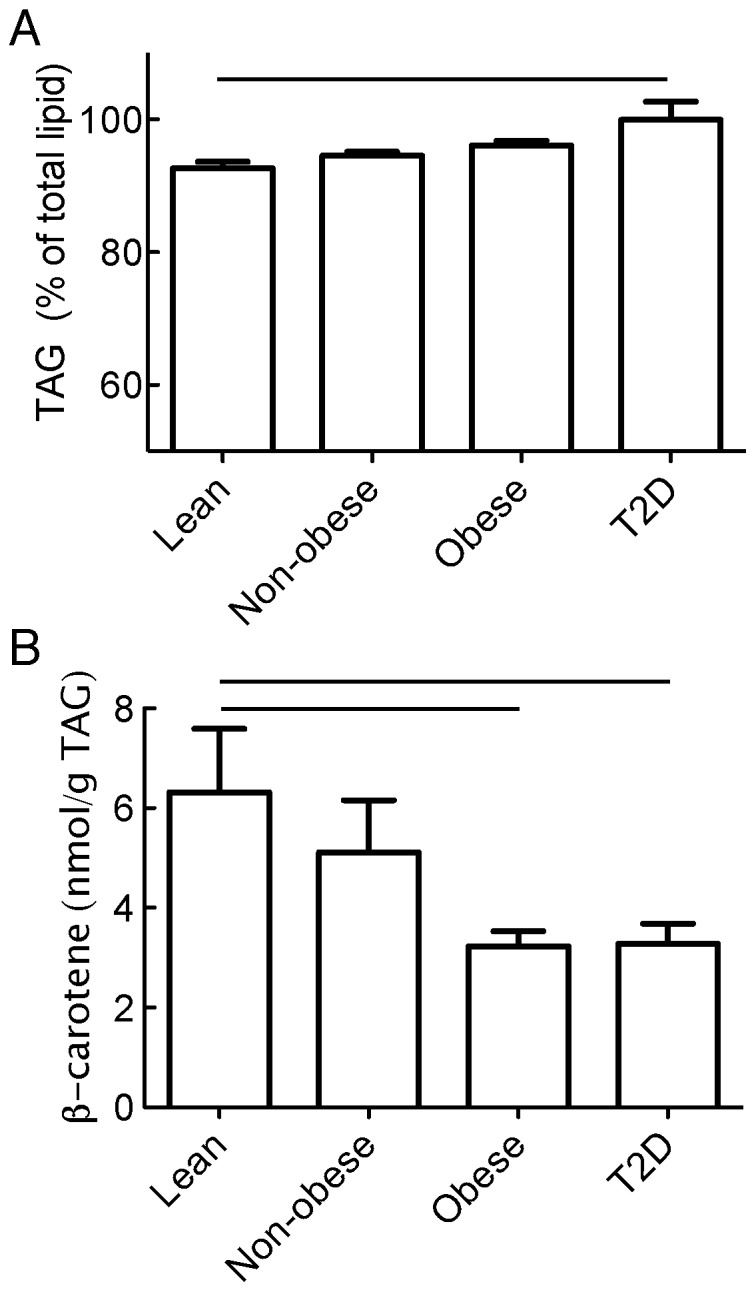
Concentration of TAG and β-carotene in lipid extracts of adipocytes. The concentrations of TAG (A) and of β-carotene (B) were determined in lipid extracts of isolated adipocytes from subjects that were divided into groups of lean (BMI<23 kg/m^2^), non-obese (23≤BMI<28 kg/m^2^), obese (BMI≥28 kg/m^2^), or obese subjects with type 2 diabetes (as indicated). Lines indicate significant differences between indicated groups (p<0.05).

We determined the concentration of β-carotene, triacylglycerol (TAG) and cholesteryl ester in the lipids extracted from isolated adipocytes obtained from the subcutaneous adipose tissues of lean, non-obese, obese and of obese type 2 diabetic women. A cut-off value of BMI (28 kg/m^2^) between the non-obese and obese groups was chosen such that the obese non-diabetic group exhibited a BMI-distribution and mean equal to the diabetic group. The obese non-diabetic and diabetic groups are thus matched with respect to their BMI and age (Table 1). The TAG fraction of all cellular lipids apparently increased in the groups with obesity, with a statistical difference between the lean and obese-T2D groups (92±1% to 99±2%, respectively) (Fig. 1A). It is likely that cellular membrane lipids (glycerophospholipids and cholesterol) constitute an important part of the difference, but these remain to identify, thus also likely explaining the higher fraction of TAG in larger and more TAG-filled adipocytes of the obese-T2D group.

**Table 1:** Subjects and clinical parameters (mean ± SD).

	Lean (BMI<23)	Non-obese (23≤BMI<28)	Obese (BMI≥28)	T2D (BMI≥28)
Number of subjects	8	11	24	12
Age (years)	61.0±8.82	66.3±13.51	54.5±14.74	55.2±15.58
BMI (kg/m2)	22.3±0.60	24.7±1.58	35.3±6.42	37.6±7.71
Cholesterol-total (mmol/L)	4.44±0.77	4.34±0.96	4.31±1.07	4.4±1.48
Cholesterol-LDL (mmol/L)	2.44±0.57	2.30±0.63	2.44±0.98	1.96±0.63
Cholesterol-HDL (mmol/L)	1.24±0.46	1.29±0.47	0.98±0.34	1.0±0.21
Triacylglycerol (mmol/L)	1.53±0.54	1.69±1.39	1.94±0.64	2.87±1.14
HOMA	2.20±1.73	2.06±0.92	1.83±1.39	–

The concentration of cholesteryl ester was in all cases below 0.1 g per 100 g of TAG (the limit of detection). Stores of cholesteryl ester are thus negligible in mature human adipocytes. This contrasts earlier findings that cholesteryl ester constitutes a substantial part (2.5–5.7%, by weight) of adipose tissue lipid stores [29], maybe reflecting cholesteryl ester storage in other cell types of the adipose tissue, but it is in line with findings that non-esterified cholesterol constitutes >90% of total adipocyte cholesterol [30].

**Figure 2:**
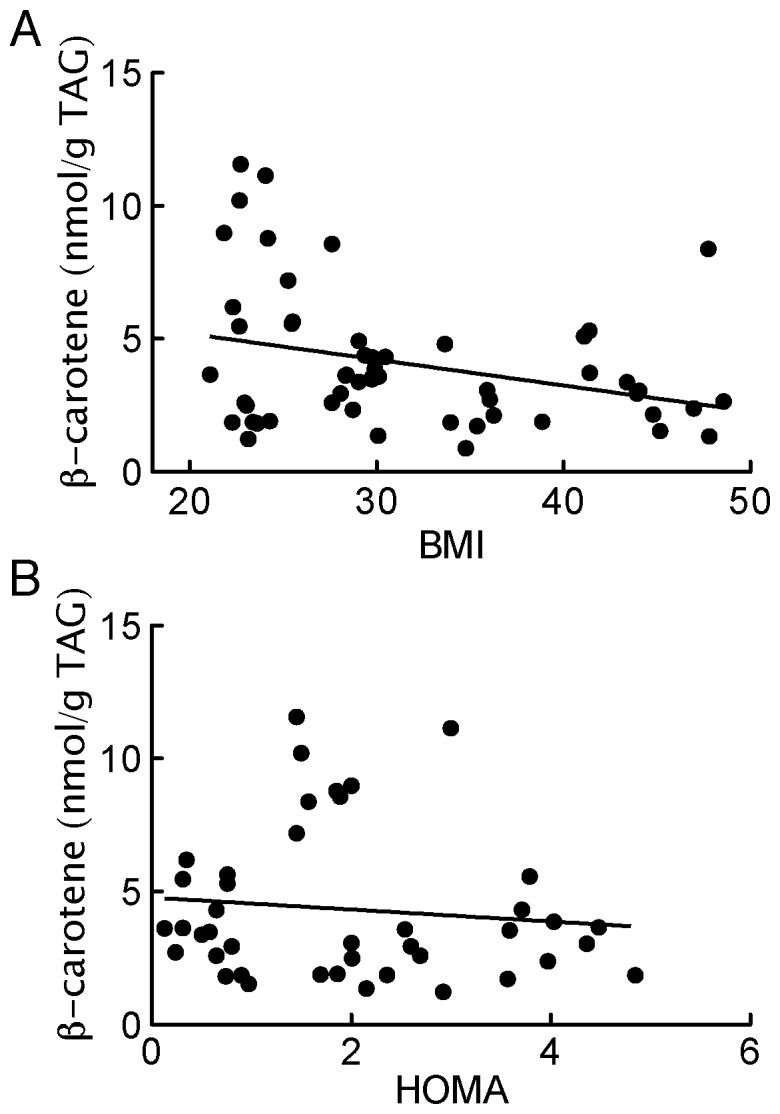
Correlation of BMI and insulin-resistance index (HOMA) with adipocyte concentration of β-carotene. (A) All subjects were included. There is a significant correlation between adipocyte content of β-carotene and BMI of donor subjects: Y = 7.14–0.10X; r2 = 0.10; p = 0.02. (B) All non-diabetic subjects were included. There is no significant correlation between adipocyte content of β-carotene and HOMA of non-diabetic donor subjects.

β-Carotene concentrations, expressed as a fraction of TAG, were substantially lower in the adipocytes from both obese non-diabetic and obese diabetic subjects compared with the lean or non-obese subjects (Fig. 1B). Moreover, β-carotene concentration of the adipocytes was correlated with BMI of donor subjects (Fig. 2A). That no difference was found between the obese non-diabetic compared with the BMI-matched obese diabetic group, indicates that the lower concentrations of β-carotene in adipocytes seen in type 2 diabetes is associated with the obesity rather than with the diabetes. This was further substantiated by lack of correlation between the individual non-diabetic subjectś adipocyte concentration of β-carotene and insulin sensitivity (Fig. 2B).

**Figure 3:**
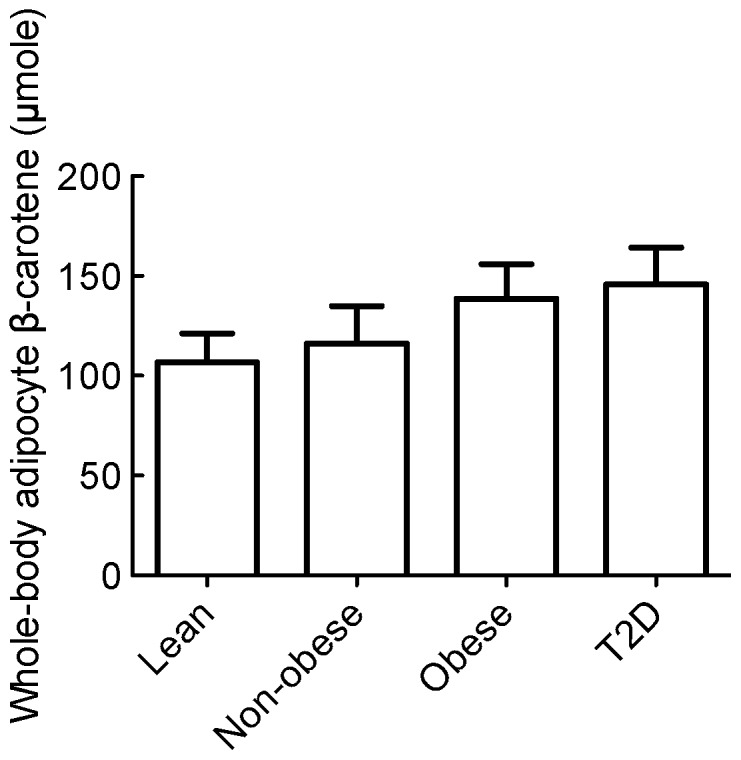
Total adipocyte stores of β-carotene in subjects. Whole body content of β-carotene stored in adipocytes was determined for each subject as the adipocyte concentration of β-carotene adjusted for total body fat. No statistically significant difference was found between the mean values in the groups using one-way analysis of variance (ANOVA), p>0.5.

Interestingly, when corrected to total body fat, the differences between the lean and non-obese subjects versus either the obese or the diabetic subjects disappeared (Fig. 3). This calculation does not take into account differences in body distribution of adipose tissue and the varied distribution of β-carotene in different adipose tissue depots, however, it suggests that whole body stores of β-carotene in adipocytes are more or less constant and maintained within a rather narrow range of 130±70 (mean±SD) µmole per person. It raises the question of how much β-carotene very lean individuals can store in their adipocytes. In our material, subjects with BMI between 21 and 23 were calculated to have about the same total amount of β-carotene as the other more obese groups (Fig. 3). It is an intriguing question how constant levels of β-carotene are maintained regardless of different BMI and varied intakes of β-carotene. Daily intake of carotenoids is probably lower on a high-energy diet than on a balanced diet with vegetables, which are the main sources of carotenoids [31]. However, intake of carotenoids has been found to correlate poorly with adipose tissue stores [23], [24], although daily intake of carotenoids has been found to inversely relate to the prevalence of metabolic syndrome [32]. A physiological indicator of adipocyte lipid stores is the adipokine leptin. Leptin is believed to be produced and released by adipocytes in proportion to the adipocyte triacylglycerol content [33]. There are no reports of effects of leptin on β-carotene content of adipocytes; although, interestingly, circulating leptin has been found to be reduced in mice by β-carotene supplementation of the diet [34].

Previous studies have found that concentrations of β-carotene in plasma are inversely correlated with measures of obesity [13], [14] and that obese subjects have reduced levels of adipose tissue β-carotene compared with lean subjects [21], [22]. Our findings herein are in accordance and show that the actual adipocyte concentration of β-carotene is inversely related to obesity. Taken together, these two observations suggest that serum levels of β-carotene may be controlled by the adipocyte stores of β-carotene, although liver also stores β-carotene. It is not known to what extent adipocyte stores of β-carotene are directly mobilized for whole-body use. It is possible that adipocytes utilize β-carotene locally for retinol synthesis or lipid stores of adipocytes act as a passive sink for excess β-carotene. In this context it is interesting that adipocytes, including those of humans, release retinol-binding protein-4 (RBP4) into the circulation, and more RBP4 is released in obesity [35]. RBP4 released from the adipocytes make cells insulin resistant by inducing the same intracellular defects as seen in type 2 diabetes [36]. Moreover, mature mouse adipocytes were found to depend completely on β-carotene for synthesis of retinoic acid and retinoic acid signaling, and retinol cannot substitute for β-carotene [37]. These findings indicate, that β-carotene is required for local synthesis of retinoic acid in the adipocytes by BCMO1, while retinol appears to be the preferred substrate in other tissues [38]. Interestingly, a diet supplemented with β-carotene, in mice, reduces body adiposity, size of adipocytes and circulating leptin levels in a BCMO1-dependent manner [34]. The concentration range of β-carotene in the lipid droplet over which β-carotene becomes rate limiting for the synthesis of retinoic acid is not known. However, knock-out of BCMO1 in mice causes β-carotene to accumulate in the adipose tissue [34]. It will be very interesting to investigate if this dependency on β-carotene for retinoic acid signaling also exists in human adipocytes.
